# Herpes Zoster in a Patient With Dengue Infection: A Case Report of a Rare Co-Infection

**DOI:** 10.7759/cureus.21310

**Published:** 2022-01-17

**Authors:** Karunakaran Samuel Rajeen, Mayurathan Pakkiyaretnam

**Affiliations:** 1 Internal Medicine, Teaching Hospital Batticaloa, Batticaloa, LKA; 2 Medicine, University Medical Unit, Faculty of Health-Care Sciences, Teaching Hospital Batticaloa, Batticaloa, LKA

**Keywords:** varicella, vesicular rash, herpes zoster, co-infection, dengue fever

## Abstract

Dengue is an infectious disease that plays an essential role in morbidity and mortality in developing countries. Occasionally, it presents with rare presentations and co-infections. Co-infection of herpes zoster and dengue is possible in countries such as these where both infections are common. Varicella-zoster infection is one of the self-limiting viral infections, and dengue fever is an endemic infection in Sri Lanka. When there is suspicion in diagnosis due to a change of natural course or overlapping of clinical features, concurrent co-infections have to be strongly suspected. We present the case of a 46-year-old female who had herpes zoster and dengue infection and was managed with a multidisciplinary team approach. The patient improved without any complications.

## Introduction

Dengue and herpes zoster infections are common viral infectious diseases in developing countries. Many co-infections with different combinations are reported with dengue fever in tropical countries such as the Chikungunya virus, hepatitis A virus, and leptospirosis [1-2]. However, diagnosing co-infections is challenging for clinicians, and life-threatening complications can occur if co-infections are not diagnosed in a timely fashion and treated appropriately. Varicella-zoster infection is one of the self-limiting viral infections. Dengue fever is an arboviral infection transmitted by mosquitoes of the genus Aedes, which is widely distributed in the tropics, including Sri Lanka [3]. Dengue infection can range from a mild viral syndrome/asymptomatic to hemorrhagic fever and shock. Though it is rare for these two viral infections to occur simultaneously, it is possible in countries such as these where both infections are common. This concomitant infection commonly occurs in the form of nonspecific febrile illness at the initial stage. Fever and rashes are the common symptoms among dengue infection and other viral illnesses, treated in primary care health settings that can cause a diagnostic dilemma in the clinical setup. We present the case of a 46-year-old female who had herpes zoster and dengue infection simultaneously.

## Case presentation

**Figure 1:**
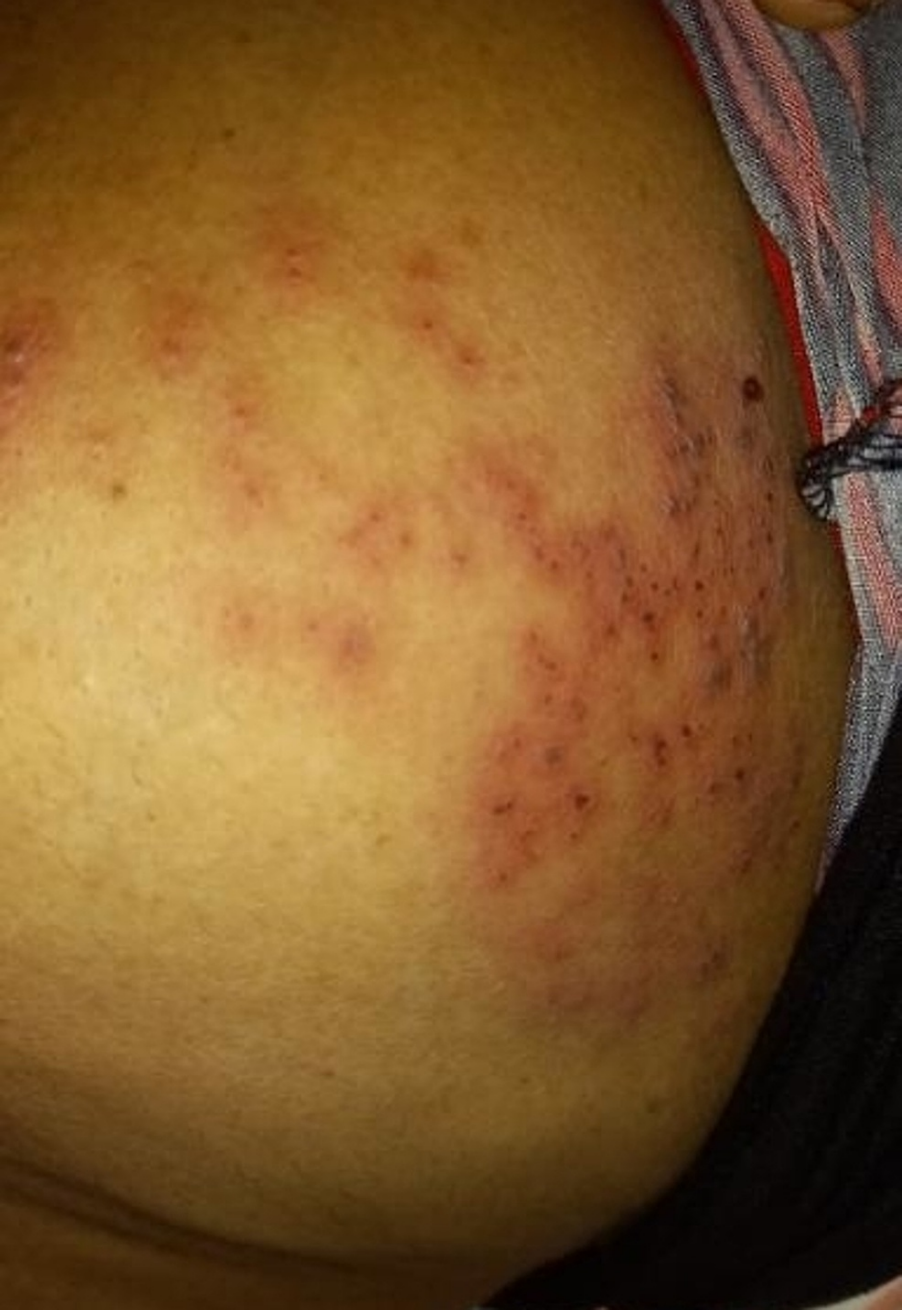
Erythematous rashes on the left side of the chest along the T4 dermatomal region Consent was obtained from the patient to publish this photograph.

A 46-year-old patient, who had not previously been evaluated medically, presented with a history of fever and rashes that she had for three days. The fever was high in nature and associated with arthralgia and myalgia. She had also had erythematous vesicular rashes on the left side of her chest from the first day of her febrile phase (Figure 1). She came from a dengue-prone area and there were reports of dengue cases from her locality.

On examination, the patient was found to be febrile, but there was no evidence of meningitis such as neck stiffness, Kernig's sign, and Brudzinski's sign - in which severe neck stiffness causes a patient's hips and knees to flex when the neck is flexed. Her blood pressure was 120/80 mmHg, her pulse rate was 96 beats per minute, respiratory rate was 22 per minute, oxygen saturation level was 100% on room air, and capillary refilling time was less than two seconds. Examination of her abdominal system revealed right hypochondrial tenderness, no hepatosplenomegaly, and no free fluids. All other systems were normal on examination, but the patient had painful erythematous vesicular rashes on the left side of her chest along the T4 dermatomal region, which became crusted over.

Her full blood count showed a white cell count of 9.7×103/µl (neutrophils 24.5%; lymphocytes 59.9%), hemoglobulin of 14.3 g/dl, hematocrit of 41.7% and platelets of 34×103/µl. Her renal function test showed serum creatinine of 57 µmol/l, blood urea of 3.6 mmol/l, serum sodium of 136 mmol/l, and serum potassium of 4.1 mmol/l. The patient’s serum glutamic oxaloacetic transaminase (SGOT) was 227U/L, her serum glutamic pyruvic transaminase (SGPT) 187 U/L, and her C-reactive protein was 20 mg/L. See Table 1 for the laboratory workup values. Her urine full report was normal, and a bedside ultrasound scan was done in the ward, which found no evidence of leakages such as no gall bladder wall edema with a thin rim of fluid around it, no fluid in the hepatorenal pouch, hepatorenal pouch, peritoneal cavity, pleural space, and no liver abnormalities. A diagnosis of dengue was made with positive serological evidence of the viral antigen detection (NS1) using the rapid test kit, which become the most common early diagnostic tool in Sri Lanka due to commercially available rapid test kits.

**Table 1:** Laboratory values Hb - hemoglobin, WBC - white blood cell count, PLT - platelets, SGOT - serum glutamic-oxaloacetic transaminase, SGPT - serum glutamic pyruvic transaminase, CRP - c-reactive protein

	On admission	Day 2	After 5 days	After 12 days	Normal range
WBC (10^9^/L)	9.7	3.67	8.39	7.5	4.0-10.0
Neutrophils	24.5%	32.4%	29.7%	42%	50%-70%
Lymphocytes	59.9%	42.3%	57.1%	32%	20%-40%
HCT	41.7%	44.2%	43.9%	40%	37%-47%
Hb (g/dL)	14.3	13.6	13.9	13.4	11.0-15.0
PLT (10^9^/L)	34	29	96	165	150-450
Blood Urea (mmol/L)	3.6	2.8	0.9	1.7	1.8-6.3
Serum Creatinine (µmol/L)	57	62	60	59	53-88
Serum Sodium (mmol/L)	136	140	142	142	136-145
Serum Potassium (mmol/L)	4.1	4.0	3.5	3.6	3.5-5.1
AST (U/L)	227	204	190	39	15-37
ALT (U/L)	187	174	170	76	12-78
C-reactive protein(mg/L)	20	14	12	3	0-5

A multidisciplinary team, which included a consultant physician, a consultant microbiologist, and a consultant, dermatologist confirmed the diagnosis of herpes zoster based on clinical findings and decided to start oral acyclovir 800 mg five times daily for five days. We managed the patient in the ward to monitor the complications of dengue.

**Figure 2:**
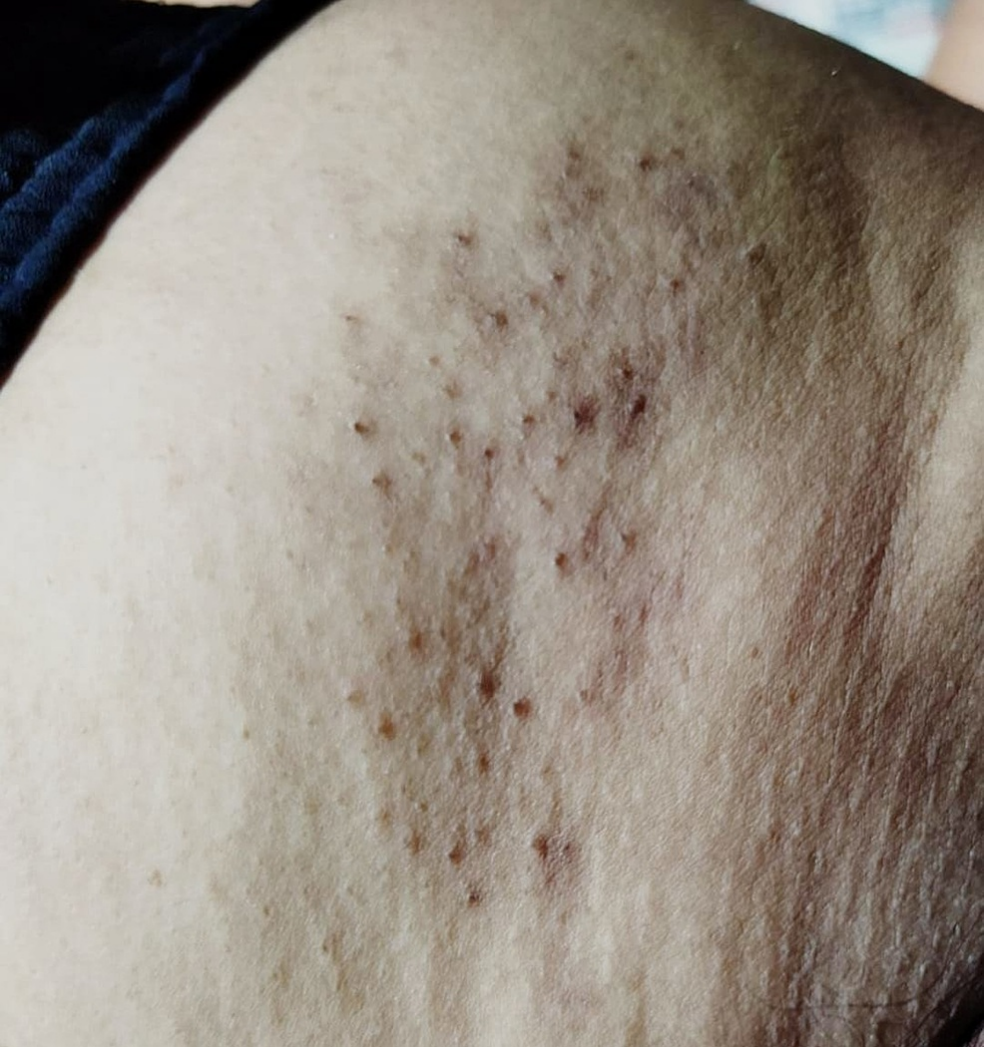
The herpetic rash has healed with areas of scarring and hypopigmentation; the hyperpigmented borders are fading out (after two months) Consent was obtained from the patient to publish this photograph.

She didn't show any evidence of dengue leakage clinically and radiological. For dengue infection, the patient was managed according to the national guidelines for dengue, with oral fluid intake (oral rehydration fluid, king coconut water, other fruit juice, rice soup, or soup rather than plain water. We excluded red and brown drinks which could cause confusion with haematemesis or coffee ground vomitus) per hour about 100 ml ( average adult of 50 kg or more - 100 ml/hour) [4]. For pain management, she was treated with carbamazepine 200 mg daily, and the pain progressively improved. The patient improved from the dengue and herpes zoster without any complications; even the rashes healed gradually (Figure 2).

She was reviewed at the ward one week later with repeated basic blood investigations, including liver transaminases, and they were within biological limits.

## Discussion

Varicella (chickenpox) is a primary infection caused by the varicella-zoster virus, which is an α-herpes virus. The varicella-zoster virus remains in the latent phase in the dorsal root ganglia. Herpes zoster infection (HZI), which is called shingles, is produced by reactivation [5]. Adults are commonly affected by HZI of the skin. The appearance of vesicles in a dermatome pattern is followed by a prodromal of deep, aching, and burning pain [6]. Postherpetic neuralgia is a commonly associated complication with herpes zoster infection in which pain persists even after the resolution of rashes for months and occasionally for years [7]. Half of the patients who are more than 50 years have the risk of developing postherpetic neuralgia [8]. Encephalitis, blindness, pneumonia, hearing problems, peripheral nerve palsies, Ramsay Hunt syndrome, Bell’s palsy, and death are complications of herpes zoster infection [9].

Dengue has global importance as a dreaded arboviral infection. It has four serotypes of epidemiological importance. The classification denotes two clinical spectrums - dengue fever (DF) and dengue hemorrhagic fever (DHF). Most cases are stereotyped and amenable to fluid resuscitation. The dengue virus (DENV) has four serotypes: DENV-1, DENV-2, DENV-3, and DENV-4. The clinical features of dengue infection include fever, arthralgia, myalgia, rashes, headache, petechiae, retro-orbital pain, spontaneous bleeding from all orifices of the body if the platelet count is severely diminished, and organ failure.

The hallmark that distinguishes DHF from DF is plasma leakage as a result of increased vascular permeability. A standard tourniquet test can be performed in the critical phase of the illness, which reflects both capillary fragility and thrombocytopenia (Table 2). Nowadays, it is used rarely because of its poor sensitivity and the availability of advanced investigations to diagnose dengue infection in Sri Lanka.

**Table 2:** Tourniquet test

Hess Capillary Resistance test (tourniquet test)
The standard blood pressure cuff is inflated at 80 mmHg for 5 minutes. If more than 10 to 20 petechiae are noted in a 1-inch square area at the cubital fossa, the test is termed positive.

Our patient presented with fever with erythematous vesicular rashes on the left side of the chest, which is a classical feature of herpes zoster. Looking at the typical rashes of herpes-zoster infection, there is a ‘cropping’ phenomenon in which all vesicles, papules, and crusting lesions are centered to a spot. Herpes-zoster rashes may resemble dengue fever rashes. The diagnosis of HZI is usually based solely on clinical presentation (unilateral, usually painful vesicular eruption with well-defined dermatomal distribution). However, in our patient, the typical presentation of painful vesicular rashes was more prominent; anyhow, we referred to a dermatologist and he confirmed the diagnosis of HZI, and the MDT decided there was no need for laboratory confirmations such as polymerase chain reaction (PCR) testing and direct fluorescent antibody testing [10].

She was evaluated for other causes of fever, including dengue infection, typhoid, and leptospirosis, which are common in Sri Lanka [2]. Since the patient comes from the dengue-prone area of Sri Lanka, we planned to exclude the dengue fever and did the basic blood investigations. In addition to that, the presence of low platelet counts and clinical features led to suspected dengue infection. We sent blood for culture, which showed no growth, and the leptospirosis antibody, immunoglobulin M (IgM), was negative; therefore, based on clinical features and the investigations' findings, we excluded leptospirosis and typhoid. Though she had a high-grade fever, MDT didn't proceed with lumbar puncture because of the low platelet count, and clinical examinations were not suggestive of meningitis. 

She was positive for NS1 antigen, which was detected by the CareStart ^TM ^Dengue Combo NS1 and IgM/IgG rapid test kit manufactured by Access Bio Inc (in the year 2021; Ewing Township, New Jersey), which has 100% specificity and 94.8% sensitivity. This serological diagnosis confirmed acute dengue infection [11]. The NS1 test is highly specific, widely used in the diagnosis of dengue fever, and cost-effective [4]. In specialized laboratories with molecular biology facilities, RT-PCR remains the gold standard of etiological confirmation. The advantage of this test is its high sensitivity and specificity on acute samples and identification of serotypes. It is, however, an expensive technology that requires sophisticated instruments and skilled professionals.

HZI usually doesn't need ward admission unless the patient is immunocompromised. In this case, as the patient had a dengue infection, and clinically, she had a febrile phase, we kept her in the ward. In addition to the management of herpes zoster, her vital parameters such as blood pressure (BP), pulse rate (PR), respiratory rate (RR), peripheral capillary oxygen saturation (SPO2), capillary refilling time, and urine output were monitored and clinically evaluated to look for features of dengue leakage and any other complications. We managed her with oral fluid and reviewed her in the ward.

Without correct diagnosis and prompt treatment, both dengue and herpes zoster can cause many severe complications. including dengue hemorrhagic shock, severe organ impairment, such as acute liver failure, acute renal failure, encephalopathy, cardiomyopathy, haemophagocytic syndrome, and non-specific peritonitis, as well as the severe complications of HZI such as encephalitis, pneumonia, and even death [9,12].

In dengue-prone areas, when a patient comes with a fever history, it is better to exclude dengue infection. However, our patient clinically improved and recovered completely without any complications. Her repeated laboratory test reports were within biological limits (Table 1).

Though dengue infection and herpes zoster don't have a direct association, dengue infection can have alterations in some autoimmune markers, such as the immune complex, which may be associated with an autoimmune-based disturbance that may trigger the reactivation of herpes zoster [13]. Anyhow, further clinical and basic studies are needed to elucidate the association between dengue infection and herpes zoster reactivation.

## Conclusions

This case report emphasizes the importance of understanding the epidemiological pattern and data of common viral diseases in our locality, their natural course, as well as the importance of appropriate clinical history-taking and examination. When there is suspicion in diagnosis due to a change of natural course or overlapping of clinical features, concurrent co-infections have to be strongly suspected. Therefore, simultaneous infection with herpes zoster and dengue has to be suspected in places where both infections are common. Delaying the diagnosis can cause severe complications since both viral infections can lead to life-threatening complications.
